# Exploratory study of an e-mentoring professional coaching model of novice midwives in Morocco

**DOI:** 10.11604/pamj.2022.41.253.29226

**Published:** 2022-03-28

**Authors:** Nabila Rouahi, Najat Boucetta, Samia Boussaa

**Affiliations:** 1 Higher Institute of Nursing and Health Techniques (*ISPITS*), Tetouan, Avenue Abdelkhalaq Torres, Saniat R´mel, 93 000 Tetouan, Morocco,; 2 Higher Institute of Nursing and Health Techniques (*ISPITS*), Ministry of Health and Social Protection, Avenue Hassan II Km 4,5 Route de Casa, 10000 Rabat, Morocco

**Keywords:** Midwifery, training of service providers, E-mentoring, E-mentor, professional competency, Morocco

## Abstract

**Introduction:**

there is a high proportion of novice midwives assigned to healthcare centers located in rural area difficult to access geographically. To improve professional communication and professionalization of these novice midwives, e-mentoring is proposed as a solution to consolidate support of the novice midwives. The aim of our research is to explore the feasibility and the acceptability of an e-mentoring professional coaching model of midwives in our geographic and professional context.

**Methods:**

an exploratory study targeted the Newly Recruited Midwives (NRMW) having less than two years of practice (N = 31) in northern Morocco. The model is inserted in a “Slack” free software where two mentors admitted the NRMW who consented to participate (N=21) in our study. The mentors ensured a synchronous and asynchronous assistance to the participants in their workplace through this platform. A questionnaire was self-administered by the participants in the first meeting. It assessed the professional skills of the participants and their coaching and training needs. The mentors shared documents and set activities in the channels of the platform. Public channels and forums have been launched. The model lasted from April 22 to May 22, 2019.

**Results:**

participation rate was 67.7% (21/31). The interactivity with the platform was uninterrupted through the period test. A high level of digital familiarization was noted. A proportion of 95% expressed the willingness to participate in an e-mentoring professional coaching model associated with a remote mentor permanently.

**Conclusion:**

the continuous interactivity of novice midwives with the e-mentoring model showed that it is feasible and acceptable in our context. Such model will solve the problems experienced by midwives in their workplace without delay or additive fees.

## Introduction

The mothers and newborns death is a global health problem. In Morocco, maternal mortality ratio is 70 per 100,000 live births and neonatal mortality rate is 13.8 deaths within 28 days per 1,000 live births [1]. Midwives play a major role in providing care for women during pregnancy and maternity [2]. The number of midwives in Morocco is 1-1.12 midwives per 10000 populations, but the need is still high [1]. There is a structured shortage of midwives. The career success is linked to formal and informal mentoring from senior to junior [3]. The mentored employees had positive outcomes career as promotion, salary and occupational status compared to employees who were not mentored [4]. Manager´s perception about psychological support in mentoring differs in terms of age whereas there is no significant difference based on gender [5]. The use of the internet for health purposes has a great potential in health promotion and reduction of social disparities in the medical sector [6]. The professional empowerment is now recognized as a mean of developing relationships, power of influence, recognition and involvement in the organization [7]. The e-coaching and the e-mentoring play a key role in continuous postgraduate development [8]. The benefits of online coaching and the reinforcement of the Mentor-Mentored relationship are obvious [9]. The added value of the electronic dimension of mentoring to the mentors is the adaptation to busy schedule and reducing student stigma [10]. E-mentoring can be more accessible in case where time and distance stands as an obstacle to the participation in mentoring model [10].

Technological development regards online mentoring as essential for its contribution to professional coaching [11], the interest and the choice of the career [12], the enhancement of positive attitudes of professionals belonging to multigenerational groups [13] and the resolution of some constraints related to geographical remoteness and labor hours [14]. Yob and Crawford set the principles of academic and psychosocial e-mentoring [15]. The capital benefit of the Information and Communication Technologies (ICT) is the coaching without traveling and other additive fees. The remote coaching via e-mentoring allows the continuous communication with the mentors and the follow-up of the mentees despite the schedule of the occupational activities of the mentors and the mentees. This can lead to the improvement of hard and soft professional skills of the mentees. The professional coaching during the first years of work increases self-confidence, transition from novice to expert, empowerment and professionalization of the midwives [16]. The disparity of the quality service between rural and urban areas is globally established. In our region, 40% of primary health care facilities are located in rural areas. The majority of our population lives in rural areas (40%). This context justifies the design of our model in this study that aimed to explore the feasibility and the acceptability of an e-mentoring professional coaching (PC) model targeting Newly Recruited MidWives (NRMW) in our region.

## Methods

**Population and ethical consideration:** we conducted an exploratory study on the feasibility and the acceptability by the NRMW to use an e-mentoring PC model in northern Morocco. The list of the eligible midwives in the region (N=31) had been provided by the responsible of the Human Resources of the regional local Health Ministry services. A midwife, recruited within the last two years and working in one of the healthcare centers of the region, is considered eligible to our study. The authorization to set this model had been previously obtained from the Ministry of Health. The study received the approval of the Ethical Committee of Mohamed V University, Faculty of Medicine and Pharmacy of Rabat (Approval N°29/20). This committee is approved by the Office of Human Research Protections.

**Model and study design:** the model was developed by two mentors working in a public institute of nursing. The first mentor was experienced in professional skills of the midwives. The second mentor was experienced in pedagogy and educational technology. They were not managers or administratively linked to any participant. They organized a meeting with the NRMW and their direct managers. All the eligible midwives of the region were invited to this meeting. The mentors were accepted by all the participants in the meeting. They explained the objective and the functioning of the model. In this meeting, a letter of invitation, consent form, information notice of participation in the e-mentoring PC model, individual password for admission into the platform to ensure an anonymous interactivity, and a self-administered questionnaire have been provided to each NRMW. Only NRMW who fulfilled the written consent (N = 21) were enrolled in the study and included in the model. At the end of the meeting, a questionnaire adapted from the e-mentoring model [15] and the official Professional Coaching Guide of midwives set on 2015 [17], was self-administered by the participants. This tool evaluates the NRMW's performance through a list of 43 competencies or skills (Table 1). It also includes items related to the socio-demographic profile of the participants, psychosocial conditions, training and support received or needed, professional motivations, labor conditions, use of the ICT, perception of professional coaching, e-mentoring and the interactivity with an online platform. These data were analyzed via Excel (version 2016).

**Table 1:** competency/skill reported in the national guide of midwives coaching

Abreviations	Competency/skill
**C1**	Management of a normal pregnancy
**C2**	Screening for high risk pregnancies
**C3**	Reference of risk pregnancies
**C4**	Bleeding management during the first half of pregnancy
**C5**	Bleeding management during the 2^nd^ half of pregnancy
**C6**	Management of postpartum bleeding
**C7**	Management of pre-eclampsia
**C8**	Management of eclampsia
**C9**	Fever management before delivery
**C10**	Fever management after delivery
**C11**	Evaluation of fetal position
**C12**	Evaluation of labor progress
**C13**	Use of the partograph
**C14**	Management of normal labor
**C15**	Management of an abnormal labor(latency phase)
**C16**	Management of an abnormal labor (active phase)
**C17**	Management of 3^rd^ phase of delivery
**C18**	Management of a labor on cicatricial uterus
**C19**	Management of a normal delivery
**C20**	Realization of vacuum delivery
**C21**	Diagnosis of a breech presentation
**C22**	Management of delivery in podalic birth
**C23**	Management of a transverse fetal position
**C24**	Management of a umbilical cord procidence
**C25**	Management of a diabetes case
**C26**	Management of a heart failure
**C27**	Realization of a amniotomy
**C28**	Realization and refection of episiotomy
**C29**	Repairing vaginal rips
**C30**	Realization of local anesthesia of the perineum
**C31**	Realization of maneuvers for shoulder dystocia
**C32**	Management of a twin delivery
**C33**	Realization of a directed delivery
**C34**	Realization of an artificial delivery
**C35**	Realization of a manual revision of uterus
**C36**	Management of a severe postpartum hemorrhage
**C37**	Realization of a care for normal newborns
**C38**	Realization of a neonatal resuscitation
**C39**	Rapid evaluation of emergency situation
**C40**	Realization of a maternal resuscitation
**C41**	Implementation of infection prevention measures
**C42**	Take care of the mother and the new born in postpartum
**C43**	Screening for anomalies in the mother and newborn

The model consists of the design by the mentors of a collaborative web-based workspace where the mentees can learn and interact in synchronous and asynchronous way with the mentors and their peers while remaining in their workplace. The mentors supply pertinent documents and organize activities in channels, forums and chats. The software adopted for the e-mentoring PC model is Slack (Slack Technologies San Francisco CA). This tool is used to improve professional communication in the field [18-21]. Slack is a free software that can store up to 10 000 messages and has a file storage capacity of 5.0 GB. It can integrate up to ten applications. The data of the mentees interactions with the platform are recorded continuously by the system and can be monitored and analyzed by the mentors. The organized workspaces are visualized in the homepage of the platform. The stack platform was installed by mentors on the mobile phone of the mentees. The participants were invited to register with their passwords. The access to the platform was restricted to the mentors (N = 2) and the consenting NRMW (N = 21). Slack software allowed the daily and weekly follow-up of the participation and the interaction of the NRMW. An integrated operating system of the platform provided an automatic recording of the data. According to this system, an active member is a member who had seen at least one content of a public channel. Private channels are not accessible in the free version of Slack software used. The platform allowed the follow-up of the parameters as the number of participants accessing, interacting and publishing content.

**Monitoring and evaluation:** the activities of coaching were set in the model through the channels and forums integrated in Slack platform. The public channels and forums organized in the platform were adapted to the needs of the NRMW notified in the questionnaire self-administered. A course channel was implemented by a set of guides and manuals on midwives competencies and professional practices. A thematic channel for Forum 1 entitled *“Pre and Post Natal Consultation” (PPNC)* was released. Another channel for Forum 2 entitled *“Emergencies in obstetrics in midwife practice”* was also inserted in the platform. A Chat and Private Mentor-Mentee communication were used. These activities allowed the participants to get answers to urgent questions in real time as well as advice in urgent situations. The channels were organized for online interactivity 7 days a week and 24 hours a day. The mentees remained connected with the mentors despite the geographic remoteness. In this workspace, they can download documents and interact in synchronous and/or asynchronous way with the mentors and the other mentees. The e-mentoring PC model lasted from April 22 to May 22, 2019. In addition to the online “Slack platform”, the mentors added an outline interactivity through a WhatsApp group, an e-mail group and Messenger.

## Results

The descriptive data of the population showed an average age of 27.26 +/- 4.12 years. Most of the participants were single and worked in rural areas (76.19%). The NRMW consent to their enrollment in the e e-mentoring PC model, based on a written consent form, was 67.7% (21/31).

**Figure 1:**
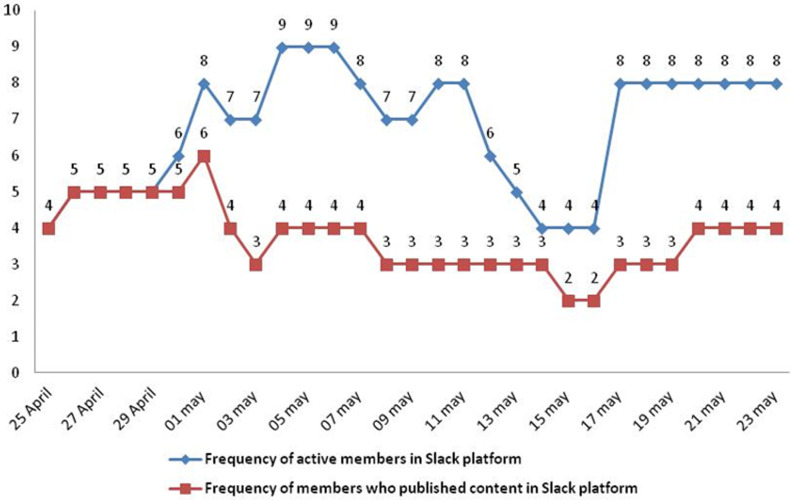
evolution of the daily participation of NRMW in Slack platform, e-mentoring PC model in Morocco, 2019

**Figure 2:**
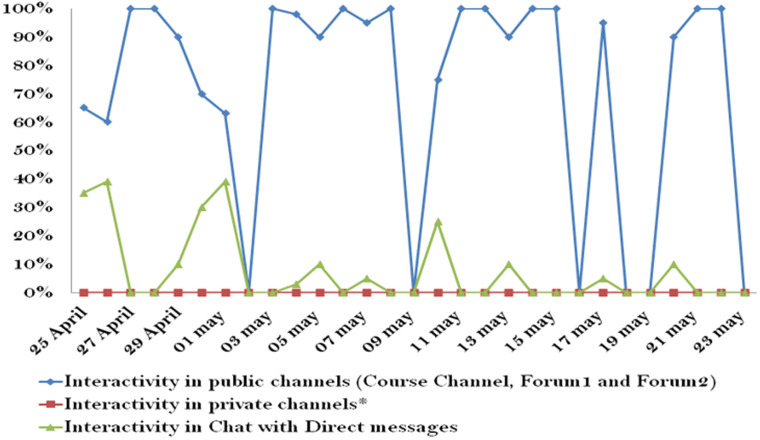
interactivity of NRMW in channels and forums of Slack platform, e-mentoring PC model, Morocco, 2019 (The free version of Slack does not give access to private channels)

**Interactivity with the model:** the online interactivity of the mentees via Slack platform supporting the e-mentoring PC model was continuous during the test period of the model (Figure 1). The daily evolution of the active members in the platform showed a variation between a maximum of 9 members and a minimum of 4 members. The access and the content publication curves showed a parallelism. The daily interactivity of active members accessing the public channel including the course and the forums reached 100% (Figure 2; S2). The interactivity in Chat reached 40%. The number of messages interchanged between the mentors and the mentees during the test period was 137. The data showed an uninterrupted interactivity of the mentees in the platform from the opening to the closing of the model. The interactivity outside the platform with the mentors counted 40 messages, 32 audio calls, 7 WhatsApp calls, 25 messages via Messenger, 14 phone calls. The topics discussed on WhatsApp are contraception, PPNC in a rural delivery, postpartum hemorrhage, placental retro hematoma, and fetal heart sound. Further topics as work management and the legal aspect of the profession practice were also discussed with the mentors.

**Familiarization with ICT and the willingness of adhesion to the model:** the questionnaire data on the use of ICT showed that all NRMW (N=21) used this technology. All the participants had a smartphone. A proportion of 57% had access to internet *“Everyday”* while 43% had access *“Few times a week”'*. All the NRMW were familiar with ICT and internet, but the nature and the duration of use varied from one participant to another. All the NRMW wished to contact the mentor *“As often as needed´*´. All the participants have no idea on a *“Professional Coaching Program”* concept and have never participated in such program. But the majority (95%) expressed the “*Willingness to participate in an e-mentoring PC model associated to a remote Coach or Mentor”*.

**Assessment of NRMW performance in professional competencies:** the data related to the evaluation of the NRMW performance in professional competencies, according to the perception of the mentees, were organized into three categories (Table 2). The category 1 includes C8, C20, C22, C23, C24, C26, C31, C36 and C40. The NRMW had a rate of mastery less than 30% for this category. Ten participants do not control any of the 9 competencies. The category 2 includes C15, C16, C25, C29, C32, C34, C35 and C39. The proportion of skilled NRMW who master these competencies was between 30 and 60%. Three NRMW do not control any of the 8 competencies. The category 3 includes C1, C2, C3, C4, C5, C6, C7, C9, C10, C11, C12, C13, C14, C17, C18, C19, C21, C27, C28, C30, C33, C37, C38, C41, C42 and C43. The proportion of skilled NRMW in this category was greater than 60%.

**Table 2:** perceptee´s performance of the NRMW (N=21), Morocco, 2019

Category	Competencies/Skills (C1-C43)	Rate of performance (%)
1	C8, C20, C22, C23, C24, C26, C31, C36, C40	[0 - 30]
2	C15, C16, C25, C29, C32, C34, C35, C39	[30-60]
3	C1, C2, C3, C4, C5, C6, C7, C9, C10, C11, C12, C13, C14, C17, C18, C19, C21, C27, C28, C30, C33, C37, C38, C41, C42, C43	≥ 60

**Constraints, needs and labor conditions:** the questionnaire data showed that a proportion of 52.38% of the participants did not receive a post-graduation course and 57.14% did not receive any training after their recruitment. All the NRMW worked in a multidisciplinary team. All the participants were accompanied by experienced midwives, 60.86% by doctors and 21.80% by gynecologists. Half of the participants hoped to be transferred to a better workplace or another city. A proportion of 43% had a workload problem, 33% had a problem in the task sharing and 5% had a communication problem. A rate of 62% of participants confirmed that they are *“Sometimes”* stressed and 5% declared that they are *“Rarely”* stressed. The first stressful item was the risk of complications with the parturient (19.04%) followed by the management of the parturient transfer to specialized structures (14.28 %). Other stressing factors like: taking responsibility, scheduling of guards, problem with staff and traditional practices of the population had been notified. Nevertheless, 57.14% of NRMW confirmed receiving *“Always”* the support of colleagues at work in difficult professional situations. A proportion of 9.52% received it *“Often”* and 33.32% received it *“Sometimes”, “Rarely”'* or *“Never”*. A proportion of 80.95% of NRMW agreed the consultation with experienced professionals. All the participants expressed their need to be accompanied by mentors.

## Discussion

Our results showed a high participation rate of the eligible population in the e-mentoring model. We noted a continuous access and interactivity of the mentees in Slack platform and the model from the opening to the closing of the test period. The data on the familiarization with ICT showed that all the participants used it. All the participants have confirmed the need to be involved in the e-mentoring model and all of them agreed to continue this experience. These data suggest the ability, the predisposition of NRMW to integrate an e-mentoring PC model and the feasibility of the model in a sustainable way. The data of the questionnaire showed that there exist a need among mentees for courses and training to strengthen their professional skills. The data revealed the need to carry out the inventory of the new required skills, the professional support by a mentor and study days centered on the specific needs of the participants as diagnosed by the questionnaire.

These data suggest the implementation of a structured intensive program based on urgent professional skills and personal skills. Such program should be implemented since the first week following the recruitment of NRMW. The midwifery team has a key role in the transition support [22]. The relationship between the midwives working alongside with the new graduates, the relationship with the team and the definition of professional boundaries, the building of a trusty relationship with the women, give the NRMW the feeling of becoming a professional midwife [23]. Other items have also been reported such as the motivation for mentoring, building strong relationships, establishing safe boundaries, managing expectations, satisfaction, retention, supporting team work and patients outcomes [24,25]. The profile of the mentor is essential for the success of a mentoring model. In our study, the election of the mentors was based on their recognized national experience in respective fields and their acceptability by the mentees. But no previous evaluation, based on specific skills of the mentors as mentioned by the authors, had been conducted. The attitudes, behaviors, knowledge and skills of the mentors are the most important factors for a successful e-mentoring model [26]. The authors described the function of the mentors in guidance, support, motivation and consideration to learners as factors that enhanced mentors’ capacity. The key elements of a mentor profile are clinical practice, education, leadership and research. A list of 24 items covering barriers and enablers of leadership enactment had been set [27]. The most important items are the healthcare system level, support from senior management, opportunity to participate at the strategic level, structural supports for the function and the size of clinical caseload. The two key elements most neglected for supporting the role of advanced practitioners are leadership and research. All these elements must be considered in the future for the implementation of an e-mentoring model for a long duration.

For the mentor, a program focusing on specific soft skills is indicated. A clinical mentor training is recommended [28]. An external assessment of the mentors’ skills conducted by peers is recommended as well [29,30]. A study conducted to assess a mentoring process through dimensions as professional support, collegiality, working levels, directivity and confidence, showed that these behaviors influence the mentoring process [31]. A structured program of experienced mentoring or peer mentoring, documented by a valid tool is advisable [32,33]. Such program should reinforce the educational level of the mentors and the mentees [34]. The model must include also a supportive culture of midwifery for a smooth transition from novice to a professional midwife [35,36].

The continuous interaction in the e-mentoring model is one of the strengths of this study. Another strengthening element is that the participants do not receive any allowance or a material motivation for interacting in the platform. The third strong point is that the mentees are not administratively linked to the mentors. Our data encourage the setting of this e-mentoring PC model in our region and test it in regions with similar geographic context in Morocco. The main limitation to the exhaustive participation of the eligible NRMW in this e-mentoring PC model, based on the mentees declarations, is the fear of being judged and fired because of misconduct in case of the dissemination of the data shared in the platform. In some rural areas, internet interruptions can compromise the synchronous activities integrated in this model. The originality and the strength of our research is that it has been conducted for the first time in Morocco and will serve as a reference for further studies in other regions and with other healthcare staff.

## Conclusion

The acceptability of the model, the admission rate in the platform and the interactivity of the mentees indicate the success of the first phase of implementation of the e-mentoring PC model in our context. The e-mentoring model continuity and sustainability is dependent on the qualifications of the mentors, the quality of the coaching and the resources shared through the platform, the satisfaction of the mentees needs and the viability of the software applied. The e-mentoring professional coaching model, developed in our study, using free software and managed by two remote mentors, is acceptable and feasible with the population of NRMW in our region.

### What is known about this topic

- In Morocco, there is no structured professional coaching program supporting the transition of midwives from novice to professional midwives;
- In our region, 40% of primary health care facilities and centers are located in rural areas; the majority of our population lives in rural areas (40%), the majority of novice midwives are assigned to centers located in rural areas;
- In Morocco and other countries, most of mother and newborn death cases occur in rural areas; this situation is linked to the unsatisfactory care and labor conditions of midwifery.

### What this study adds

- This study demonstrates that this model is feasible and acceptable by novice midwives in our context; therefore, all of the participants express their willingness to participate in such model continuously;
- The participants express clearly their need to have a remote mentor who provides psychological and professional support in the workplace without additive fees related to face-to-face coaching (travel and living fees);
- Our data show that continuous follow-up of the participants by the mentors is perceived as necessary; this need is linked to the fact that a novice midwife can face difficult situations at any time in their workplace therefore; she has no sufficient experience to solve it alone.
